# Mammaglobin B is an independent prognostic marker in epithelial ovarian cancer and its expression is associated with reduced risk of disease recurrence

**DOI:** 10.1186/1471-2407-9-253

**Published:** 2009-07-27

**Authors:** Renata A Tassi, Stefano Calza, Antonella Ravaggi, Eliana Bignotti, Franco E Odicino, Germana Tognon, Carla Donzelli, Marcella Falchetti, Elisa Rossi, Paola Todeschini, Chiara Romani, Elisabetta Bandiera, Laura Zanotti, Sergio Pecorelli, Alessandro D Santin

**Affiliations:** 1 Division of Gynecologic Oncology, Department Materno Infantile e Tecnologie Biomediche, University of Brescia, Brescia, Italy; 2 Section of Medical Statistics and Biometry, Department of Biomedical Sciences and Biotechnology, University of Brescia, Brescia, Italy; 3 Department of Pathology, University of Brescia, Brescia, Italy; 4 Department of Obstetrics & Gynecology, Division of Gynecologic Oncology, Yale University New Haven Hospital, CT, USA

## Abstract

**Background:**

Traditional prognostic factors in epithelial ovarian cancer (EOC) are inadequate in predicting recurrence and long-term prognosis, but genome-wide cancer research has recently provided multiple potentially useful biomarkers. The gene codifying for Mammaglobin B (MGB-2) has been selected from our previous microarray analysis performed on 19 serous papillary epithelial ovarian cancers and its expression has been further investigated on multiple histological subtypes, both at mRNA and protein level. Since, to date, there is no information available on the prognostic significance of MGB-2 expression in cancer, the aim of this study was to determine its prognostic potential on survival in a large cohort of well-characterized EOC patients.

**Methods:**

MGB-2 expression was evaluated by quantitative real time-PCR in fresh-frozen tissue biopsies and was validated by immunohistochemistry in matched formalin fixed-paraffin embedded tissue samples derived from a total of 106 EOC patients and 27 controls. MGB-2 expression was then associated with the clinicopathologic features of the tumors and was correlated with clinical outcome.

**Results:**

MGB-2 expression was found significantly elevated in EOC compared to normal ovarian controls, both at mRNA and protein level. A good correlation was detected between MGB-2 expression data obtained by the two different techniques. MGB-2 expressing tumors were significantly associated with several clinicopathologic characteristics defining a less aggressive tumor behavior. Univariate survival analysis revealed a decreased risk for cancer-related death, recurrence and disease progression in MGB-2-expressing patients (p < 0.05). Moreover, multivariate analysis indicated that high expression levels of MGB-2 transcript (HR = 0.25, 95%, 0.08–0.75, p = 0.014) as well as positive immunostaining for the protein (HR = 0.41, 95%CI, 0.17–0.99, p = 0.048) had an independent prognostic value for disease-free survival.

**Conclusion:**

This is the first report documenting that MGB-2 expression characterizes less aggressive forms of EOC and is correlated with a favorable outcome. These findings suggest that the determination of MGB-2, especially at molecular level, in EOC tissue obtained after primary surgery can provide additional prognostic information about the risk of recurrence.

## Background

Epithelial ovarian cancer (EOC) accounts for the majority of ovarian malignancies and is estimated to be the third most common malignancy of the female genital tract and the first leading cause of death from gynecological cancer in the US in 2008 [1]. The paucity of disease specific symptoms and the lack of effective clinical markers contribute to the high mortality rate of EOC. Although primary surgical cytoreduction and standard care, based on first-line chemotherapy with carboplatin and paclitaxel, successfully induce clinical remission in about 70% of the cases, prognosis remains poor for as many as 50% of the patients who will experience recurrence and die of secondary disease within 5 years after diagnosis. The pathological stage, the histological grade, the bulk residual tumor and the tumor histology constitute the most important prognostic factors and aid clinical decision-making in patients with EOC [2,3]. Nevertheless, their value in predicting recurrence and long-term prognosis remains inadequate [4]. Despite the efficacy of adjuvant chemotherapy in patients with primary ovarian cancer, occurrence of relapse is one of the major problem in EOC management. So, it is of the utmost importance to discover new potential prognostic biomarkers that may allow stratification of patients into meaningful prognostic categories, with the ultimate goal of improving treatment strategies following primary debulking surgery.

Several new genes involved in epithelial ovarian carcinogenesis have been identified by high-throughput transcription profiling techniques such as high density oligonucleotide microarrays. By analyzing the genetic fingerprints of 19 ovarian serous-papillary carcinomas (OSPCs), our research group has recently identified Mammaglobin B (secretoglobin, family 2A, member 1 – SCGB2A1) as the top differentially expressed gene in OSPCs compared to normal human ovarian surface epithelium (HOSE) cell controls [5]. Mammaglobin B gene was first isolated by Becker in 1998 [6] and codifies for a small secreted protein of the uteroglobin superfamily that includes nine human secretoglobins localized on chromosome 11q12.2 [7]. Normal expression of MGB-2 has been described in secretory mucosal epithelia of breast [6], uterus [6,8] and lacrimal glands [9] and it is likely to be hormonally induced by androgens, as found in human ocular tissues [9], prostate [10], and pituitary [11]. Apart from the ovarian adenocarcinomas, abnormal expression of MGB-2 has been observed in epithelial tumors originating from breast [12-14], lung [15], digestive organs [16] and biliary tract [17]. Our previous study demonstrated the wide expression of MGB-2 at mRNA and protein level on multiple histological types of EOC, especially in endometrioid tumors [18]. A following study on type I endometrial cancers with endometriod histology reported a significantly inverse correlation between MGB-2 expression and tumor grade that is the main prognostic factor for this neoplasm [19].

Since currently available clinical markers are not completely satisfactory for the prognostic evaluation of patients with EOC, in the present study we extended our molecular and immunohistochemical MGB-2 expression findings previously reported in ovarian cancer [18] and analyzed MGB-2 in a larger cohort of clinically well characterized EOC patients. The final goal of this study was to investigate MGB-2 association with clinicopathological features and to assess MGB-2 prognostic value.

## Methods

### Patients and tissue samples

This study was performed on 106 consecutive cases of epithelial ovarian cancer diagnosed and treated at the Division of Gynecologic Oncology at University of Brescia, Italy, between September 2003 and July 2006. Study approval was obtained from the Institutional Review Board and all patients signed an informed consent according to institutional guidelines. No patient received preoperative chemotherapy or radiotherapy. All specimens examined in this study were collected at primary surgery. Optimal debulking surgery, defined as no macroscopic residual disease at the end of the procedure, was achieved in 42 out of 106 (40%) patients, while the remaining patients had a residual tumor (TR) greater than 0.5 cm in diameter. The age of patients ranged from 24 to 88 years, with a median age of 60 years. Histological subtype and differentiation grade were assigned according to World Health Organization (WHO) criteria and were further reviewed by two experienced pathologists. The staging procedure was performed according to the International Federation of Gynecology and Obstetrics (FIGO) system standards. Clinical and pathological features are shown in Table 1. For survival analysis, patients were stratified in "early-stage" tumors (stage IA to IIB, 25 cases) and in "advanced-stage" tumors (stage IIC to IV, 81 cases). After surgery, most patients (96 out of 106, 90%) received a first line platinum-based chemotherapy. Among the remaining 10 patients, 4 were not eligible for adjuvant treatment, 2 received a non platinum-based chemotherapy, 1 refused adjuvant therapy and 3 did not receive chemotherapy because of their poor medical condition. Patients were followed up from the date of surgery until death or September 30, 2008 (median follow-up, 30.5 months, range 1 – 78 months). Clinical data were collected from medical records and were available for all the patients. At the time of the last follow-up, 45 (43%) patients were alive without evidence of disease, 12 (11%) patients were alive with disease and 49 (46%) patients had died of disease (median OS, 49 months, CI_95% _= 33 - ∞).

**Table 1:** Patients characteristics

*Clinicopathological features*	*n*	*%*
**Age (median, range) ys**	60 (24–88)	
≤ 40	12	12%
> 40	94	88%
**Histological type**		
clear-cell	11	10%
endometrioid	19	18%
undifferentiated	5	5%
mixed	14	13%
mucinous	3	3%
serous-papillary	54	51%
**FIGO stage**		
≤ IIB	25	24%
> IIB	81	76%
**Histological grade**		
G1	7	7%
G2	13	12%
G3	86	81%
**Residual tumor (TR), cm**		
TR = 0	42	40%
TR ≥ 0.5	64	60%
**Ascites**		
yes	62	58%
no	44	42%
**Lymph nodal involvement**		
negative	53	50%
positive	28	26%
missing	25	24%
**Preoperatory CA125 level**		
< Threshold**	11	10%
≥ Threshold**	92	87%
missing	3	3%

**Threshold = 200 U/ml for pre-menopausal patients & 35 U/ml for post-menopausal patients.

### Ovarian specimens

Briefly, 106 ovarian tumor tissues and 27 normal ovarian samples from women who underwent oophorectomy for uterine fibromas or prolapse were enrolled in the study. Median age of the control group was 55 years (range, 48 to 71 years).

Tissues were identified, sharp-dissected and snap-frozen in liquid nitrogen within 30 minutes from resection. All tissue fragments were split for histological confirmation. The samples were embedded in optimal cutting temperature (O.C.T.) medium, microdissected and the frozen sections were stained with hematoxylin and eosin (H&E) to check epithelial component. Pathological examination confirmed the absence of disease on normal ovarian biopsies. Out of the 106 epithelial ovarian cancer samples studied, 98 contained at least 70% neoplastic epithelial cells and were retained for further total RNA extraction. All the 27 normal ovarian controls were eligible for RNA extraction and were represented by 7 ovarian surface epithelium brushings, 10 ovarian surface epithelium (HOSE) primary cell lines and 10 surgical biopsies. Ovarian surface epithelium brushings were obtained with a sterile cytology brush from the normal ovaries of donors. The brush was first touched to a glass slide and then immediately immerged in a solution that preserves RNA quality (RNA*later*^®^, Applied Biosystems, Applera UK, Cheshire, UK). The slide was later stained following a modified Papanicolaou protocol to confirm epithelial content.

### Establishment of HOSE primary cell lines

A total of 10 HOSE primary cell lines were established after sterile processing of samples from surgical biopsies. HOSE were derived from normal ovarian epithelial tissue of patients undergoing surgery for benign pathologies. Pathological examination confirmed the absence of any neoplastic disease. To obtain pure HOSE short term cell cultures, the normal ovarian tissue was macrodissected and incubated in 2 ml collagenase and DNAse for 30 minutes at 37°C with occasional agitation. Sheets of HOSE cell fragments were gently scraped with a rubber scraper directly into complete growth medium M199/MCDB105 (Invitrogen Life Technologies, Carlsbad, CA, USA/Sigma, St. Louis, Missouri, USA) supplemented with 10% fetal bovine serum, 200 μg/ml penicillin and 200 μg/ml streptomycin. Primary cell lines were maintained in the same complete growth medium at 37°C, 5% CO2 in tissue culture 6-well plates (Corning, NY, USA) and used to generate monolayers. Total life of in vitro culture was less then 14 days for all samples. Normal cell cultures were collected for RNA extraction at 70–80% confluence without being subcultured (passage 0). The epithelial purity of normal ovarian cell lines were evaluated by immunocytochemical staining with antibody against pan-cytokeratin and epithelial membrane antigen (EMA) as previously described [20]. All the cell cultures were composed of at least 99% epithelial cells and were retained for RNA extraction.

### Total RNA extraction and reverse transcription

Total RNA was obtained from 98 cancer samples including 76 primary ovarian tissues with different histologies and 22 omental metastases, and from three types of normal controls: 10 HOSE primary cell lines, 10 ovarian biopsies and 7 ovarian brushings. One hundred mg of frozen tissue were sharply dissected from each sample and homogenized with a rotary homogenizer (QIAGEN, Valencia, CA, USA) in RNA Lysis Solution (Invitrogen Life Technologies). Total RNA was prepared from tissues, cells and brushings using the PureLink Micro-to-Midi Total RNA Purification System (Invitrogen Life Technologies). Purity and RNA quantity were evaluated spectrophotometrically. The RNA integrity was tested on Agilent 2100 Bioanalyser (Agilent Technologies, Santa Clara, CA, USA) and only RNA samples having an OD 260/280 ratio > 1.8 and an Integrity Number > 8.5 were retained for further amplification. For the generation of first-strand cDNA, 1 μg of total RNA was reverse-transcribed using random hexamers in a final volume of 20 μl according to the SuperScriptTM II RT RNaseH-Reverse Transcriptase protocol (Invitrogen Life Technologies).

### Quantitative-RealTime-PCR

Real-time polymerase chain reaction was performed in duplicate by using primer set and probe specific for MGB-2 gene. All the reactions were carried out on the ABI PRISM 7000 Sequence detection System (Applied Biosystems) using the TaqMan Universal PCR master Mix and the following Assay on Demand (Applied Biosystems): Hs00267180_m1 (Mammaglobin B). One μl of the reverse transcription volume was used for each PCR reaction in a total volume of 25 μl. The thermal cycling conditions were the following: 10 min at 95°C, 40 cycles of denaturation at 95°C for 15 sec and annealing-extension at 60°C for 1 min. The comparative threshold cycle (CT) method was used for the calculation of amplification fold as specified by the manufacturer. Commercially available primers and probe for GADPH mRNA were used for normalization (Applied Biosystems). Mammaglobin B mRNA quantities were analyzed in duplicate and mean CT levels were used for further analyses. Results were normalized against GADPH and expressed in relation to a calibrator sample. Results per PCR reaction were expressed as relative gene expression, using the delta-delta CT method [21]. The calibrator was chosen among normal controls and was given a relative expression value of 1.

### Immunohistochemistry on formalin-fixed tissues

To evaluate MGB-2 protein expression level, immunohistochemical staining (IHC) was performed on 106 neoplastic samples including 84 primary tumors and 22 omental metastases, and on 10 normal ovaries, stored in the Department of Pathology at the University of Brescia, Italy. Formalin-fixed, paraffin-embedded tissues were cut and stained with Hematoxilin and Eosin (H&E) and analyzed by a Staff Surgical Pathologist. As controls, surface epithelia obtained from normal ovaries were used. Briefly, formalin-fixed, paraffin-embedded tissues were cut at 2 μm, mounted on charged slide, and dried. For immunohistochemical analysis, slides were deparaffinized and rehydrated in graded solutions of ethanol and distilled water. Endogenous peroxidase was blocked by incubation with peroxidase-blocking solution (DAKO ChemMate, CA, USA) for 15 minutes, followed by rinsing in tris-buffered saline (TBS). Non-specific staining was blocked by treatment with normal goat serum (1:50) for 5 minutes. The immunohistochemical method involved sequential application of primary antibody to Mammaglobin diluted 1:50 (Mammaglobin Rabbit Monoclonal Antibody (clone 31A5), Zeta Corporation, Sierra Madre, CA, USA) for 45 minutes, a secondary biotinylated anti-rabbit antibody diluted 1:20 (Menarini, Florence, Italy) for 15 minutes and streptavidin-biotin complex diluted 1:20 (Reagent kit, Menarini) for 15 minutes. The immunoprecipitate was visualized by treatment with 3'3-diaminobenzidine (Bio-optica, Milan, Italy) for 5 minutes and counterstained by hematoxylin (DAKO ChemMate). Immunostaining was considered positive for MGB-2 when at least 10% of neoplastic cells were stained. All samples were scored quantitatively and qualitatively in 20 and 40 high power fields in every section (Nikon, Eclipse E400).

### Evaluation of immunohistochemistry

All immunohistochemically stained slides were examined on a multiheaded microscope by two pathologists experienced in EOC, who were blinded to patient outcome. Both staining extent and staining intensity were considered for evaluation and a four-tiered scoring system was used: 0 for negative cases, 1+ for weak, 2+ for moderate, 3+ for strong immunoreactivity. Then, the raw data were dichotomized as follows: the low (1+), the moderate (2+) and the high (3+) MGB-2 expressing cases were grouped for statistical analysis and assigned the designation 1, whereas the completely negative cases were designated as 0.

### Statistical analysis

The association between MGB-2 mRNA expression and clinicopathologic parameters was investigated with an ANOVA using the MGB-2 mRNA relative quantification value on log scale. The association between IHC, expressed as binary variables code as score = 0 or score ≥ 1, and clinical covariates were evaluated by means of a Fisher's exact test or logistic regression.

For survival analysis, three endpoints (cancer relapse, cancer progression and death due to cancer) were used to calculate Disease-Free Survival (DFS), Progression-Free Survival (PFS) and Overall Survival (OS), respectively. DFS was defined as the time interval between the date of surgery and the date of identification of disease recurrence (37 events), PFS was defined as the time interval between the date of surgery and the date of identification of progressive disease (disease not treatable with curative intent, 53 events) and OS was defined as the time interval between the date of surgery and the date of death (49 events). For all three endpoints the last date of follow-up was used for censored subjects. Survival analyses were all performed using the Cox proportionals hazard model, while survival curves were plotted using the Kaplan-Meier method. To evaluate the effect of MGB-2 mRNA expression on prognosis (DFS, PFS or OS) we first divided MGB-2 mRNA relative quantification values in terziles, computed on the whole set of data, then compared the first two terziles (Low & Medium) to the highest one (High). The prognostic significance of MGB-2 expression at protein level was assessed considering immunostaining as a binary variable, coded as IHC = 0 or IHC ≥ 1.

Median survival was defined as the time-point where the KM curves crossed the horizontal line at 50% survival probability. That is at the time where at least 50% of the patients in the specific group experienced the event of interest.

For all endpoints, the independent contribution of MGB-2 on cancer prognosis was evaluated using two approaches. First we fitted survival models accounting for all the clinicopathologic variables known to be associated with prognosis plus MGB-2. This would evaluate the contribution of MGB-2 on survival corrected for all clinical covariates (irrespective of their significance in our data). As some clinical variables had a substantial number of missing data, we performed multiple imputation (m = 10) using the MICE algorithm [22].

Second, a model selection of survival fit was performed using bootstrap resampling in conjunction with stepwise procedure based on Akaike criterion (AIC) [23]. The rationale of the procedure is based on the fact that automated selection procedures are likely to identify noisy variables. Bootstrap can help identify truly independently associated variables [24]. We performed the variable selection based on a combination of missing data multiple imputation (MI) and bootstrap resampling, thus allowing control for both the variability in the imputation as well as in the automated selection procedure [24]. Multiple imputation was performed based on the MICE algorithm with 10 imputation steps, and each imputed dataset was bootstrapped using 50 replications, for 500 stepwise procedures overall. Variables that were selected in at least 70% of the replications were included in the final model.

All the analyses were considered significant at p ≤ 0.05. All the analyses were performed with the statistical software R, version 2.8.0 [25].

## Results

### Mammaglobin B gene expression in ovarian cancer tissues and in normal ovarian controls

Mammaglobin B gene expression was tested by qRT-PCR in 98 neoplastic samples represented by 76 primary EOC specimens with various histologies and 22 serous omental metastases. MGB-2 expression in tumors was compared with three sources of normal cells, including 10 HOSE primary cell lines, 7 ovarian brushings and 10 ovarian biopsies. The optimal cutoff point was determined by means of a Receiver Operating Characteristic (ROC) curve and it was set at relative quantification, RQ = 2560. According to the chosen threshold, 24 out of 27 normal controls were classified as negative (specificity = 88.9%), while 87 out of 98 EOCs were classified as positive (sensitivity = 88.8%). A highly significant increase in MGB-2 expression was found in tumors compared to normal controls (Mann-Whitney test, p < 0.001). Relationships between MGB-2 expression and clinicopathologic parameters are illustrated in Table 2. MGB-2 expression was significantly lower in primary serous tumors (p = 0.036), serous metastasis (p < 0.01) and undifferentiated (p < 0.01) tumor compared to endometrioid histotype. Mammaglobin-B expression significantly decreased with advancing stage (expression in stage ≤ IIB ~5 times higher than in stage > IIB, p < 0.01), increasing residual disease (TR > 0 cm vs TR = 0 cm, p = 0.034), ascites production (p = 0.017) and elevated preoperative CA125 serum level (p = 0.026). Despite well-differentiated tumors showed higher MGB-2 mRNA expression than moderately and poorly differentiated ones, pairwise differences between both G3 and G2 versus G1 cases for MGB-2 relative gene expression values were not statistically significant (Table 2).

**Table 2:** Association between MGB-2 mRNA expression and clinicopathologic variables in 98 patients.

Parameter	Fold change	95%CI	p-value
**Age at diagnosis (y)**			
> 40 vs ≤ 40	1.60	0.38 – 6.62	0.517
			
**Histological type**			
clear-cell vs endometrioid	0.47	0.09 – 2.40	0.361
undifferentiated vs endometrioid	0.05	0.01 – 0.44	< 0.010
serous metastases vs endometriod	0.14	0.04 – 0.53	< 0.010
mixed vs endometriod	0.28	0.06 – 1.23	0.090
mucinous vs endometrioid	0.34	0.03 – 4.08	0.387
serous vs endometrioid	0.26	0.07 – 0.91	0.036
			
**FIGO stage**			
> IIB vs ≤ IIB	0.19	0.07 – 0.51	< 0.010
			
**Histological grade**			
G2 vs G1	0.47	0.04 – 4.99	0.642^$^
G3 vs G1	0.22	0.03 – 1.68	0.159^$^
G2&G3 vs G1	0.32	0.04 – 2.61	0.337^$^
			
**Residual tumor (TR), cm**			
TR ≥ 0.5 vs TR = 0	0.38	0.16 – 0.93	0.034
			
**Ascites**			
yes vs no	0.37	0.16 – 0.83	0.017
			
**Lymph nodal involvement**			
positive vs negative	0.53	0.19 – 1.49	0.228
			
**Preoperative CA125 serum level**			
≥ Threshold vs < Threshold**	0.19	0.04 – 0.81	0.026

** Threshold = 200 U/ml for pre-menopausal patients & Threshold = 35 U/ml for post-menopausal patients.

$ corrected for multiple testing.

### Expression patterns of Mammaglobin B protein in normal ovary and in epithelial ovarian cancer by immunohistochemistry

**Figure 1:**
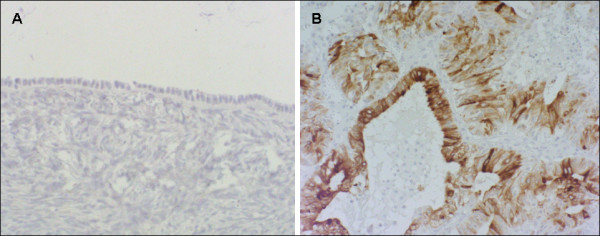
**Representive immunohistochemical staininig for MGB-2 in normal ovary and ovarian adenocarcinoma**. Celomic epithelium and parenchyma of normal ovary are negative for MGB-2 (A). Early-staged ovarian adenocarcinoma with endometrioid histology showing a staining intensity variable from moderate to strong (B).

**Figure 2:**
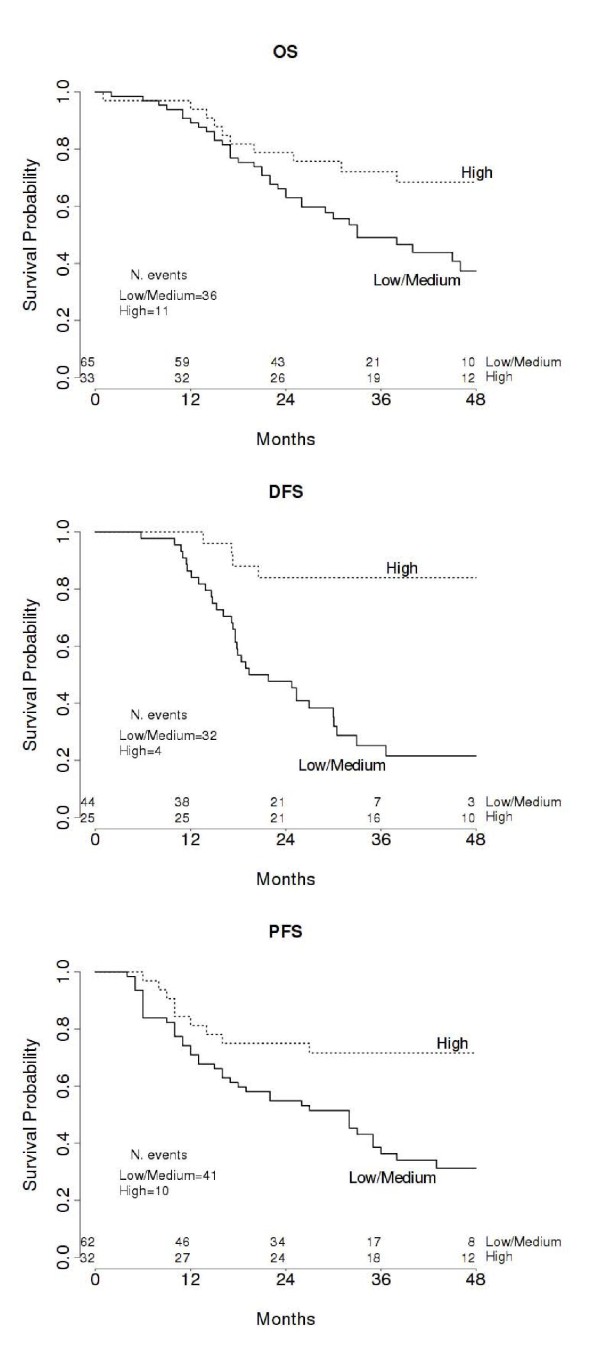
**Univariate survival analyses plots**. Kaplan Meier analyses showing the pattern of Overall Survival (A), Disease-Free Survival (B) and Progression-Free Survival (C) relative to MGB-2 mRNA expression. Number of events and patients at risk every 12 months are displayed.

The analysis of 10 normal ovaries showed that MGB-2 was not constitutively expressed in the celomic epithelium or in the stroma (Figure 1A) or ciliated epithelium lining inclusion cysts (data not shown). Of the 106 EOC specimens examined in this study, 42 (40%) cases were positive for MGB-2 immunoreactivity: 39 out of 84 (46%) primary ovarian cancers, and 3 out of 22 (14%) omental metastases. A variable expression of MGB-2 was observed among different tumor histotypes: the protein was undetectable in most of the serous tumors (44 out of 54, 81%) independently from their primary or metastatic origin, as well as in mucinous (2 out of 3, 67%) and undifferentiated adenocarcinomas (4 out of 5, 80%). On the contrary, most of the endometrioid (15 out of 19, 79%), clear-cell (7 out of 11, 64%) and mixed tumors (8 out of 14, 57%) stained positively for the protein. In particular, a strong immunoreactivity was observed in 5 tumors: 3 with pure (Figure 1B) and 2 with mixed endometrioid histology. The staining was restricted to epithelial cancer cells and localized in the cytoplasm with a diffuse and granular pattern, while reactive stromal cells adjacent to ovarian tumor cells were found negative in all the pathologic samples analyzed (Figure 1B). Reduced MGB-2 protein expression was significantly associated with tumor histology (p < 0.001), advanced FIGO stage (p < 0.001), suboptimal debulking (p = 0.004), presence of ascites (p = 0.010), and lymph nodal involvement (p = 0.056) (Table 3). A non-significant trend toward reduced expression of MGB-2 protein in less differentiated tumors was observed.

**Table 3:** Association between MGB-2 protein expression and clinicopathological parameters.

		MGB-2 protein expression		
	n	score = 0	score ≥ 1	P*
		n (%)	n (%)	
Total	106	64 (60.4)	42 (39.6)	
				
**Age at diagnosis (y)**				
≤ 40	12	6 (50.0)	6 (50.0)	
> 40	94	58 (61.7)	36 (38.3)	
				0.535
**Histological type**				
endometrioid	19	4 (21.1)	15 (78.9)	
clear-cell	11	4 (36.4)	7 (63.6)	
undifferentiated	5	4 (80.0)	1 (20.0)	
serous-pap.met.	22	19 (86.4)	3 (13.6)	
mixed	14	6 (42.9)	8 (57.1)	
mucinous	3	2 (66.7)	1 (33.3)	
serous-papillary	32	25 (78.1)	7 (21.9)	
				<0.001
**FIGO stage**				
≤ IIB	25	7 (28.0)	18 (72.0)	
> IIB	81	57 (70.4)	24 (29.6)	
				<0.001
**Histological grade**				
G1	7	2 (28.6)	5 (71.4)	
G2	13	6 (46.2)	7 (53.8)	
G3	86	56 (65.1)	30 (34.9)	
				0.092
**Residual tumor (TR), cm**				
TR = 0	42	18 (42.9)	24 (57.1)	
TR ≥ 0.5	64	46 (71.9)	18 (28.1)	
				0.004
**Ascites**				
no	44	20 (45.5)	24 (54.5)	
yes	62	44 (71.0)	18 (29.0)	
				0.010
**Lymph nodal involvement**				
negative	53	27 (50.9)	26 (49.1)	
positive	28	21 (75.0)	7 (25.0)	
missing	25	16 (64.0)	9 (36.0)	
				0.056
**Preoperatory CA125 level**				
< Threshold**	11	4 (36.4)	7 (63.6)	
≥ Threshold**	92	58 (63.0)	34 (37.0)	
missing	3	2 (66.7)	1 (33.3)	
				0.205

*Fisher exact test.

**Threshold = 200 U/ml for pre-menopausal patients & 35 U/ml for post-menopausal patients.

#### Relationship between MGB-2 protein expression and tumor histology

Next, we evaluated the association between MGB-2 coded as binary (IHC = 0; IHC ≥ 1) and single histotypes by means of a logistic regression, with p-value correction for pairwise multiple comparisons. The odds ratio (OR) of expressing MGB-2 protein was significantly higher in endometrioid tumors than in primary (OR = 1.76, p_adjusted _< 0.001) and metastatic serous EOCs (OR = 1.93, p_adjusted _< 0.001). Moreover, the probability of observing MGB-2 protein expression in serous metastases was lower than in mixed (OR = 0.64, p_adjusted _= 0.039) and clear-cell (OR = 0.60, p_adjusted _= 0.022) primary EOCs. A good correlation was found between MGB-2 mRNA levels and protein scores (n = 98, r = 0.54, p < 0.001, Spearman correlation).

### Survival analysis

#### Relationship between MGB-2 expression at mRNA and protein level and overall survival in EOC patients

In the univariate analysis for overall survival, high expression of MGB-2 mRNA and immunohistochemical score 1+ were significantly correlated to longer survival, as well as the other traditional prognostic factors (Table 4). The median OS for the low-medium MGB-2 mRNA expressing group (65 patients, 36 events) was 33 months (95%CI, 26 to ∞), while it was not defined for the high MGB-2 mRNA expressing group (33 patients, 11 events). The median OS for the MGB-2 protein expressing group (42 patients, 12 events) was not defined, while it was 38 months (95%CI, 26 to 60) for the negative group (64 patients, 37 events). The Kaplan Meier survival curve shown in figure 2A confirmed that patients with tumors expressing high levels of MGB-2 had a prolonged overall survival. When MGB-2 was entered into a multivariate model along with FIGO stage, age, grade, residual tumor, ascites, preoperative CA125 serum level and lymph nodal involvement, it didn't show any significant effect (Table 4). Similarly after automatic model selection only FIGO stage was kept in the final model (selected in 94% bootstrap samples) and addition of MGB-2 to the model delivered no significant improvement (Table 4).

**Table 4:** Univariate and multivariate survival analyses in relation to MGB-2 and clinical parameters.

	OS				DFS				PFS			
Variables	N	HR	95% CI	p	N	HR	95% CI	p	N	HR	95% CI	p
***Univariate analysis***												
												
**MGB-2mRNA RQ**												
high vs medium & low	98	0.45	0.22–0.87	0.016	69	0.15	0.05–0.37	< 0.001	94	0.36	0.17–0.69	0.002
**MGB-2 immunostaining**												
1+ vs 0	106	0.47	0.24–0.87	0.015	75	0.28	0.12–0.58	< 0.001	102	0.43	0.22–0.77	0.004
**Age**												
> 40 vs ≤ 40	106	1.98	0.77–7.23	0.172	75	2.51	0.84–12.27	0.107	102	1.72	0.73–5.28	0.234
**FIGO stage**												
≤ IIB vs >IIB	106	0.06	0.007–0.24	< 0.001	75	0.13	0.03–0.35	< 0.001	102	0.05	0.006–0.20	< 0.001
**Residual tumor**												
TR ≥ 0.5 vs TR = 0	106	4.62	2.31–10.41	< 0.001	75	2.34	1.22–4.69	0.010	102	4.11	2.17–8.54	< 0.001
**Presence of ascites**												
yes vs no	106	2.64	1.44–5.13	0.001	75	2.66	1.37–5.45	0.003	102	2.52	1.42–4.70	0.001
**Lymph nodes**												
positive vs negative	82	2.93	1.42–6.13	0.004	65	2.93	1.44–5.89	0.004	82	2.43	1.27–4.62	0.008
**Grade**												
G2&G3 vsG1	106	2.95	0.79–26.15	0.118	75	3.04	0.81–27.08	0.110	102	3.17	0.86–28.01	0.092
**Preoperative CA125 serum level**												
≥ Threshold vs < Threshold*	103	2.80	0.95–13.62	0.064	74	4.87	1.30–43.35	0.014	99	3.15	1.07–15.30	0.035
												
Multivariate analysis**												
***A*.**												
**MGB-2mRNA RQ**												
high vs medium & low	106	0.79	0.38–1.62	0.52	75	0.25	0.08–0.75	0.014	102	0.63	0.30–1.31	0.22
B.												
**MGB-2 immunostaining**												
1+ vs 0	106	1.02	0.51–2.04	0.96	75	0.41	0.17–0.995	0.048	102	0.89	0.46–1.73	0.76

Abbreviations: HR, hazard ratio; CI, confidence interval of the estimated HR.

*Threshold = 200 U/ml for pre-menopausal patients & 35 U/ml for post-menopausal patients.

**Multivariate models were adjusted for stage of disease, grade, age, presence of ascites, residual tumor and lymphatic involvement. Multiple imputation data.

#### Relationship between MGB-2 expression at mRNA and protein level and disease free survival in EOC patients

Univariate analysis for DFS in the subgroup of 75 patients showed again that high MGB-2 mRNA expression and score 1+ were favourable prognostic factors, along with all the other known prognostic factors (Table 4). The median DFS for the low-medium MGB-2 mRNA expressing group (44 patients, 32 events) was 20.6 months (95%CI, 17.3 to 30), while it was not defined for the high MGB-2 mRNA expressing group (25 patients, 4 events). The median DFS for the MGB-2 protein expressing group (32 patients, 8 events) was not defined, while it was 25.3 months (95%CI, 17.6 to 30.5) for the negative group (43 patients, 29 events). The Kaplan Meier survival curve for high versus low-medium expression of MGB-2 mRNA is shown in figure 2B. There was a significant difference in DFS for tumors with high versus low-medium expression of MGB-2 gene. The favorable effect of MGB-2 on time to recurrence was also found when MGB-2 was evaluated by immunohistochemistry. In the full model accounting for all clinical covariates MGB-2 mRNA expression was still highly significant, suggesting an independent contribution on patients relapse-free survival (HR = 0.25, p = 0.014). Consistently MGB-2 positive immunostaining showed a reduction in the hazard (HR = 0.41, p = 0.048) (Table 4). Model selection allowed for inclusion of MGB-2 mRNA expression (selected in 96% of bootstrap samples) along with FIGO stage (selected in 88% of bootstrap samples). When considering MGB-2 score instead of RQ in the same multivariate model, MGB-2 along with FIGO stage and ascites presence were selected in the final model (MGB-2, FIGO stage and ascites presence were selected in 81%, 77% and 70% of bootstrap samples, respectively).

#### Relationship between MGB-2 expression at mRNA and protein level and progression free survival in EOC patients

In the subgroup of 53 patients affected by progressive disease, those with high MGB-2 mRNA expressing tumors or positive immunostaining experienced a longer progression-free survival time, as indicated by Cox regression analysis (Table 4) and represented by the Kaplan Meier curve (Figure 2C). The median PFS for the low-medium MGB-2 mRNA expressing group (62 patients, 41 events) was 32 months (95%CI, 16 to 36) while it was not defined for the high MGB-2 mRNA expressing group (32 patients, 10 events). The median PFS for the MGB-2 protein expressing group (41 patients, 13 events) was not defined, while it was 32 months (95%CI, 16 to 43) for the negative group (61 patients, 40 events). However, the pathological stage was the only independent predictor of cancer progression in the multivariate model (FIGO stage selected in 97% of the bootstrap samples), and neither MGB-2 mRNA nor immunostaining were associated to PFS (Table 4).

#### Time dependent ROC

Given the possible interest of MGB-2 mRNA expression as a clinical marker for prognosis, we tried to identify a reasonable cutoff that might account for disease-free survival time. To do this, we fitted a time-dependent ROC curve from censored survival data [26] using a predicted time point at 36 months. Briefly, this method computes ROC curves based on a time-dependent status indicator (i.e. relapse in our case) accounting for censored survival information. The optimal MGB-2 mRNA cutoff was approximately RQ ≈ 32768, corresponding to 68.1% sensitivity and 75.3% specificity (AUC = 75.3%).

## Discussion

Mammaglobin B is a secretoglobin family member, known to be normally expressed by mammary gland [6], human endometrium [8] and pituitary [11] and to be involved in the development of adenocarcinomas originating from various organs [12-17], including ovary and endometrium, as recently described by our group [18,19]. Mammaglobin B is considered as a useful candidate marker for the molecular detection of minimal residual disease in lymph nodes [12-14,27,28] and for the diagnosis of occult tumor cells in effusions from patients with various malignancies including those harboring gynecological cancers [29]. Even though MGB-2 function remains unknown, its wide-spread overexpression in multiple histological types of EOC compared to normal celomic epithelium and its presumably secretory nature suggest that it could be an attractive diagnostic and prognostic biomarker for ovarian malignancy [18]. Previous studies have shown an association between the expression of Mammaglobin A (MGB-1), which is highly homologous to Mammaglobin B, and less aggressive breast cancer phenotype, providing independent prognostic information for cancer patients' survival outcomes [30-32]. Since traditional prognostic factors are imperfect predictors of clinical outcome in epithelial ovarian cancer [4], in this study we have analyzed the potential prognostic impact of MGB-2 expression on EOC patient survival.

Although histological subgroups were somehow limited in their sample size, our current study provides evidence of a significant MGB-2 up-regulation at both mRNA and protein level in ovarian cancer, specifically in the endometrioid subtype. These results differ from our previous study [18], where, likely due to the small number of samples available for statistical analysis, significant differences in MGB-2 expression were not detected amongst the different histological types of ovarian cancer. Other than histology, MGB-2 expression was found significantly associated with traditional clinicopathological parameters indicating favorable prognosis, such as early FIGO stage, optimal debulking success, absence of ascites, absence of lymph nodal involvement and normal preoperative CA125 serum level. In this regard, although FIGO stage includes lymph nodal status and positive cytology, these parameters have also been analyzed separately in our study because of their ascertained clinical prognostic relevance [3]. In our previous study on endometrioid endometrial cancer, a significant inverse relationship between MGB-2 expression and differentiation grade had been demonstrated [19]. Unexpectedly, present investigation shows a non-significant relationship between MGB-2 expression and histological grade, both at mRNA and protein level. However, this lack of association may be due to the imbalanced number of patients available for the analyses in the three subgroups (G1, G2, G3).

Finally, we investigated the potential prognostic value of MGB-2 expression on patient outcome. Since follow-up data were available for all 106 patients, survival analysis was performed on the entire group of ovarian cancer patients. In the univariate analysis, MGB-2 expression was significantly correlated with reduced risks of cancer-related death, recurrence and disease progression. As shown by Kaplan Meier plots, significantly longer overall survival and disease-free survival were observed in high MGB-2-expressing patients compared to those with low/medium MGB-2 expression as well as in patients showing positive immunostaining when compared to patient harboring MGB-2 negative tumors. Similarly, a significant improvement in time to progression was identified for MGB-2 expressing patients, both in terms of mRNA and protein levels. When a multivariate survival model accounting for the effect of MGB-2 expression in relation to all the other established prognostic indicators was applied, MGB-2 expression, especially at mRNA level, retained its independent prognostic significance for disease-free survival along with FIGO stage, whose prognostic role in EOC is well established [2,3]. It is noteworthy that MGB-2, although showing higher expression in ovarian cancer with endometrioid histology when compared to other histologic types, maintained its independent prognostic value for DFS in the multivariate analysis.

In our study two different techniques (i.e., RT-PCR and IHC) were used to evaluate MGB-2 expression. In this regard, tumor marker quantification by real-time PCR is well known to be less error-prone and much more accurate than IHC, since with the latter technique, negative histological results may not be representative of the entire tissue status. Unfortunately, however, although MGB-2 determination at mRNA level might be a potentially useful prognostic marker for planning therapy and follow-up, to date molecular techniques such as real-time PCR, are not used in routine clinical practice for ovarian pathology.

Limited information is currently available regarding MGB-2 biological function or factors regulating its expression in human tumors. In this regard, recent studies have ruled out a potential "growth factor" activity of its analog MGB-1 on tumor cells as MGB-1 ectopic expression in breast tumor cell lines didn't affect their cellular proliferation rate in vitro [33]. Furthermore, for Uteroglobin (UG), the founding member of the secretoglobin superfamily [34], and to date, the only secretoglobin extensively studied, tissue-specific expression in the uterus and oviduct has been shown to be regulated by several steroid hormones and to be enhanced by prolactin [34]. Among its multiple biological properties, UG binds hydrophobic ligands, such as progesterone and prostaglandins, and seems to play a homeostatic role against oxidative damage, inflammation, autoimmunity and cancer [34]. Importantly, regardless to its yet poorly understood function in cancer, our MGB-2 expression results, although heterogeneous among the ovarian cancer tissues tested, clearly showed that the highest MGB-2 expression correlated with favorable clinicopathologic features and reduced risk of relapse. These findings are therefore consistent with the results reported for the overexpression of its homologous MGB-1 in breast cancer patients [30,31]. Indeed, in this patient population, a prolonged DFS and an association with favorable prognostic clinical variables, including positive estrogen and progesterone receptors' status has previously been reported [30-32].

## Conclusion

Our findings support MGB-2 as a novel prognostic marker in EOC. Its expression status is tightly associated with clinicopathologic tumor variables affecting prognosis and is prominent in early-stage and in endometrioid tumors. Since MGB-2 has shown an independent prognostic value for future recurrence, its determination in EOC tissue samples could be clinically useful in the attempt to identify patients at different risk of relapse before starting standard chemotherapy and, subsequently, to optimize follow up and further adjuvant treatment. Further studies on a larger patients' cohort are warranted to validate the prognostic impact of MGB-2 expression on survival.

## Abbreviations

MGB-2: human Mammaglobin B; EOC: epithelial ovarian cancer; HOSE: human ovarian surface epithelium; qRT-PCR: quantitative Reverse Transcription-Polymerase Chain Reaction; IHC: immunohistochemistry; H&E: hematoxilin and eosin; OS: overall survival; DFS: disease-free survival; PFS: progression free survival; HR: hazard ratio; CI: confidence interval; OR: odds ratio; MI: multiple imputation.

## Competing interests

The authors declare that they have no competing interests.

## Authors' contributions

ADS conceived, coordinated, designed the study, interpreted the data and revised the manuscript. RAT carried out the molecular expression study, reviewed medical records, designed the database, interpreted the data, drafted and wrote the report. SC performed statistical analyses, figures and tables, interpreted the data and helped to draft the manuscript. AR supervised the research group and critically reviewed the manuscript. EB helped in collecting data from medical records, carried out RNA extraction and critically reviewed the manuscript. FEO and GT conceived the study, designed the clinical database, helped in reviewing medical records, interpreted the data and critically reviewed the manuscript. CD and MF performed pathological and immunohistochemical study. ER coordinated the immunohistochemical study. PT and LZ helped in collecting follow-up data. CR and EB participated in collecting flash-frozen tissue samples and in establishing HOSE primary cell cultures. SP provided funds and participated in the design of the study. All of the authors have read and approved the final manuscript.

## Pre-publication history

The pre-publication history for this paper can be accessed here:

http://www.biomedcentral.com/1471-2407/9/253/prepub
